# Modulating mitochondrial quality in disease transmission: towards enabling mitochondrial DNA disease carriers to have healthy children

**DOI:** 10.1042/BST20160095

**Published:** 2016-08-15

**Authors:** Alan Diot, Eszter Dombi, Tiffany Lodge, Chunyan Liao, Karl Morten, Janet Carver, Dagan Wells, Tim Child, Iain G. Johnston, Suzannah Williams, Joanna Poulton

**Affiliations:** * Nuffield Department of Obstetrics and Gynaecology University of Oxford, The Women's Centre, Oxford OX3 9DU, U.K.; † Institute of Reproductive Sciences, University of Oxford, Oxford OX3 9DU, U.K.; ‡ School of Biosciences, University of Birmingham, Birmingham, U.K.

**Keywords:** mitochondrial replacement therapy, mitophagy, mtDNA bottleneck

## Abstract

One in 400 people has a maternally inherited mutation in mtDNA potentially causing incurable disease. In so-called heteroplasmic disease, mutant and normal mtDNA co-exist in the cells of carrier women. Disease severity depends on the proportion of inherited abnormal mtDNA molecules. Families who have had a child die of severe, maternally inherited mtDNA disease need reliable information on the risk of recurrence in future pregnancies. However, prenatal diagnosis and even estimates of risk are fraught with uncertainty because of the complex and stochastic dynamics of heteroplasmy. These complications include an mtDNA bottleneck, whereby hard-to-predict fluctuations in the proportions of mutant and normal mtDNA may arise between generations. In ‘mitochondrial replacement therapy’ (MRT), damaged mitochondria are replaced with healthy ones in early human development, using nuclear transfer. We are developing non-invasive alternatives, notably activating autophagy, a cellular quality control mechanism, in which damaged cellular components are engulfed by autophagosomes. This approach could be used in combination with MRT or with the regular management, pre-implantation genetic diagnosis (PGD). Mathematical theory, supported by recent experiments, suggests that this strategy may be fruitful in controlling heteroplasmy. Using mice that are transgenic for fluorescent LC3 (the hallmark of autophagy) we quantified autophagosomes in cleavage stage embryos. We confirmed that the autophagosome count peaks in four-cell embryos and this correlates with a drop in the mtDNA content of the whole embryo. This suggests removal by mitophagy (mitochondria-specific autophagy). We suggest that modulating heteroplasmy by activating mitophagy may be a useful complement to mitochondrial replacement therapy.

## The problem of heteroplasmic mtDNA disease and pre-implantation development

**Figure 1:**
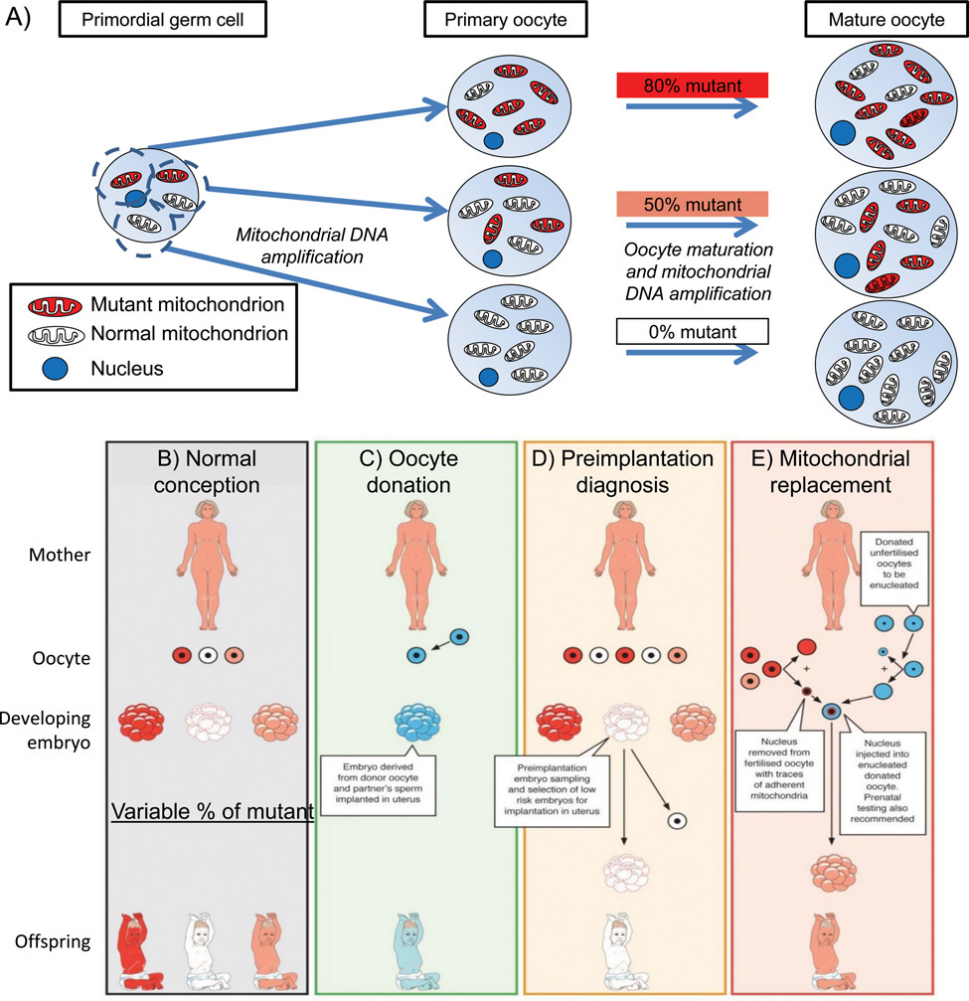
Transmission of mtDNA disease and strategies to prevent transmission of mtDNA mutations (**A**) Cartoon of the mitochondrial bottleneck: heteroplasmy of mtDNA in primordial germ cells may segregate during the ∼50-fold increase in mtDNA content as they develop into primary oocytes, resulting in different mutant loads (0–80% in this illustration). Although a major component of the trans-generation switching in mutant load has occurred by the oocyte stage, further segregation occurs during embryonic and fetal life. Three available ways to reduce the risk of transmitting mitochondrial DNA disease: oocyte donation, pre-implantation genetic diagnosis and mitochondrial replacement therapy. Red represents mutant mitochondrial DNA, pink and white represent successively higher proportions of normal mitochondrial DNA. Blue represents genetic material from an unrelated donor. (**B**) No intervention: offspring's mutant mitochondrial DNA load will vary greatly. (**C**) Oocyte donation: current availability in the United Kingdom is limited by the availability of oocyte donors. (**D**) Pre-implantation genetic diagnosis: is available in the United Kingdom for most mitochondrial DNA diseases. (**E**) MRT nuclear transfer: being developed in the United Kingdom, first cases likely this year, not yet available in the United States.

Mitochondrial diseases range from severe to very mild and common. These potentially affect up to one in 400 individuals, all of whom are likely to develop impaired hearing, but very few severe complications [1]. Those caused by pathogenic mutations in mtDNA are problematic because of the unique biology of maternal inheritance. Prenatal diagnosis [2–4] and even estimates of risk are fraught with uncertainty [5] because of heteroplasmy (co-existing normal and mutant mtDNA). There is also a threshold effect in most mtDNA diseases, with the level of heteroplasmy required for symptoms to become manifest varying from <10% to 100% mutant mtDNA in different tissues. In addition, there is an mtDNA bottleneck whereby dramatic and unpredictable fluctuations in the proportions of mutant and normal mtDNA may arise between generations (illustrated in Figure 1B): recent work combining mathematical theory and experiments has helped elucidate the debated mechanism of this process [6]. During transmission of mtDNA from mother to child, significant fluctuations are already apparent in oocytes in both controls [7] and carriers of mtDNA disease [8–10].

The clinical implications of the mitochondrial bottleneck and the strategies for preventing transmission of mtDNA disease are illustrated in Figure 1. Oocyte donation (Figure 1C) is an appropriate strategy for all maternally transmitted mtDNA disease because it effectively reduces the risk of transmission to the population prevalence. Pre-implantation genetic diagnosis (PGD, Figure 1D) [2] is widely licensed to reduce transmission of mtDNA diseases from mother to offspring [11]. In PGD the mutant load of embryos produced by *in vitro* fertilization (IVF) is estimated from either 1–2 cells taken from cleavage stage embryos, or approximately five trophoblast cells from blastocysts cultured *in vitro.* If the embryo with the lowest mutant mtDNA load is selected for transfer to the uterus, this will greatly reduce the risk and severity of mtDNA disease in any resulting pregnancy [3,12]. In practice, some centres set a threshold that depends on the penetrance of the mutation, above which transfer will not be performed. Estimating mtDNA mutant load from a single blastomere of a cleavage stage embryo [13] is accurate. Measurements based on trophoblast cells in a blastocyst biopsy have been successful [14] but are more controversial [15,16]. This might be because mtDNA segregation coincides with the increase in oxidative phosphorylation that occurs at implantation.

Mitochondrial replacement therapy (MRT, Figure 1E) is now available in the UK as an alternative approach to PGD, apparently successful in monkeys [17] and mice [18], and imminently to be performed in humans. Introducing the maternal nucleus into a donor cell with healthy mtDNA immediately before (metaphase spindle transfer [17]) or after (pronuclear transfer, along with the male pronucleus [19]) fertilization is more effective in increasing the proportion of normal mtDNA [10]. However, there are difficulties in synchronizing menstrual cycles [20], risks from imprinting of nuclear DNA [21] and from compatibility between nuclear and mitochondrial DNA [22] as well as ethical concerns around having three genetic parents [23].

‘Cytoplasmic transfer’ of donated, healthy mitochondria has been applied clinically with a view to improving function in aged human oocytes. There is some evidence that oocytes that are depleted of mtDNA benefit from this treatment in pigs [24]. In humans however, an ongoing study that has been widely publicized [25] is controversial [26]. Given that this technique aimed to supplement and not replace the mother's mitochondria, it is not surprising that only a low level of injected mtDNA was detectable in the resulting ‘transmitochondrial’ children [27]. However, one transmitochondrial child born after cytoplasmic transfer was held to be autistic [28], but the numbers were insufficient to determine whether this procedure caused any overall long-term problems to the children.

One potential complication arising from these therapies is the risk of introducing non-compatible mtDNA [22], so that mtDNA segregation favours the pathogenic mutant mtDNA. To analyse this issue, Burgstaller et al. [22] produced four heteroplasmic mouse models by ooplasm transfer, placing various naturally occurring mtDNA haplotypes from mice captured from the wild in Europe on to a common laboratory mouse mtDNA and nuclear background (C57BL/6N). The wild-derived haplotypes used differ considerably from each other and from C57BL/6N, leading to variable genetic distances between haplotypes in the four crosses. A mathematical framework facilitated the direct comparison of many of these mice, revealing that tissue-specific segregation was very common (including within post-mitotic tissue types), the magnitude of segregation increasing with the genetic distance between the mtDNA haplotypes [22]. These data suggest that unpredictable segregation of mutant mtDNA could impair the effectiveness of mitochondrial replacement therapy unless donor and recipient mtDNA haplotype are closely matched [29]. This would be of particular concern if heteroplasmy *per se* were in some way detrimental [30].

Another promising approach to reducing the load of pathogenic mutant mtDNA in the germline involves transcription activator-like effector nucleases (TALENs) [31]. These can be targeted to mitochondria to cleave different classes of pathogenic mtDNA mutations. TALENs have high specificity for the mutant being targeted, and this approach is sufficiently versatile to target many different mutations. It can be adapted for use in germ cells [32]. Current problems are that the mtDNA copy number is knocked down by the procedure by perhaps 75% of the starting level, to a level rather close to the threshold number of mtDNAs required for successful embryonic development.

## Mitophagy improves mitochondrial quality

**Figure 2:**
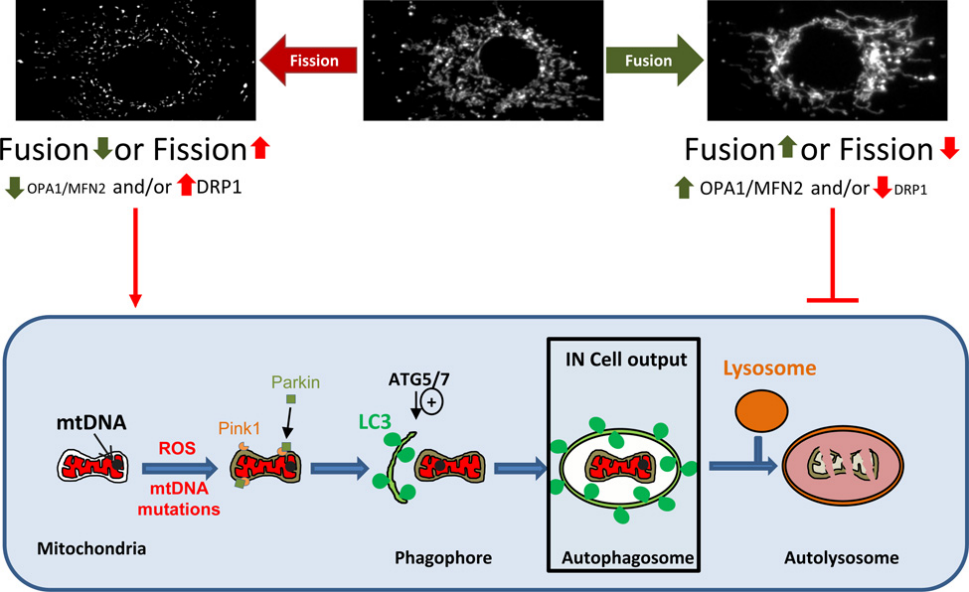
The mitophagy pathway The mitochondrial network is dynamic with events of membrane fusion and fission within the network. The morphology of the mitochondrial network observed reflects the balance between these events. An increase in fission or a decrease of fusion is favourable to mitophagy when an increase of fusion or decreased fission inhibits the mitophagy. A simplified view of mitophagy is represented in the cartoon; briefly a dysfunctional mitochondria is targeted, potentially by Parkin and Pink1 proteins, to a forming autophagosome, the phagophore. The phagophore formation is under the control of Atg5/7 proteins notably. The autophagosome matures and engulfs the mitochondria before fusing with a lyososome for degradation. Our IN Cell system is able to measure the co-localization between mitochondria and autophagosome.

Mitophagy is a mitochondria-specific type of autophagy (self-degradation by cells) with the potential to remove mtDNA mutants, illustrated in Figure 2. Mitophagy is regulated by both mitochondrial membrane potential and dynamics. In the best-known description, mitochondrial depolarization activates PINK1 to recruit ubiquitin ligase, Parkin, to mitochondria, leading to clearance. On the other hand excessive mitochondrial fission leads to depolarization-independent mitophagy [33]. This may be exacerbated by reduced expression or ubiquitinylation of pro-fusion proteins OPA1 and the mitofusins (Mfn1 and Mfn2) or activation of pro-fission proteins such as DRP1. Damaged mitochondria are recruited to the autophagosome via the mitochondrial proteins Nix and BNIP3 and the adapters P62 and LC3-II. Autophagy proteins ATG5 and ATG7 regulate formation of the autophagosome. The autophagosome then fuses with a lysosome and its contents degraded at low pH. Small regions of the mitochondrial network serviced only by detrimental mutant mtDNA may have a decreased membrane potential and may be isolated by fission [34]. The clearance of these non-functional, isolated mitochondria could filter out damaged mtDNA preventing transmission to the next generation [35–39].

Although widely discussed, this type of mitophagy may be quantitatively less important than mitophagy that is activated by nutrient deprivation. These processes have been designated type 2 and type 1 mitophagy respectively [40]. Type 1 mitophagy declines with age [41] and may remove the bulk of reactive oxygen species (ROS) that result from oxidative phosphorylation. It could underlie the beneficial effect of low nutrient intake on longevity [42]. Type 2 mitophagy involves PINK1 and Parkin and appears to be important in preventing neurodegeneration. Whereas dysfunctional mitochondria often fragment, other types of mitochondrial energetic stress, including nutrient deprivation and exposure to the mitochondrial poison doxorubicin [43], induce fusion of the network, variously described as mitochondrial ‘elongation’ [44] and ‘stress-induced mitochondrial hyper-fusion’ (SIMH) [45]. This pro-survival mitochondrial response to stress prevents mitophagy [44] and increases cellular ATP, probably enabling cells to tolerate high levels of mutant mtDNA [46]. While an adaptive response in the short term, SIMH prevents mitochondrial fragmentation and may impair mitochondrial quality control by mitophagy, as well as playing other physical and chemical roles in cells [47]

Autophagy is essential for normal pre-implantation development in mice [48]. Embryos lacking oocyte-specific expression of Atg5 and hence normal autophagosomes failed to develop beyond the 4–8-cell stage [49]. Although mitophagy could be critically important for controlling mtDNA segregation at this stage, little or nothing is known about mitophagy in the germline.

## Measuring mitophagy

Studies of mitophagy are less prominent that one might expect, given its potential for modifying the course of mtDNA disease. This may be because both autophagy and mitophagy are transient processes and hence difficult to measure. The abundance and flux of autophagosomes engulfing mitochondria are specific measures of mitophagy that are preferred to quantifying key autophagy proteins [50]. We therefore not only measured the number of autophagosomes and mitochondria engulfed by autophagosomes, but also used mtDNA analysis to assess mitochondrial quality. As mitophagy events are rare, we chose to use high-throughput microscopy [41] to ensure that a large number of cells are analysed. The mitochondrial quality analysis was based on the observation that the mutation m.3243A>G is progressively lost when the cells are grown in conditions requiring oxidative metabolism (glucose-free galactose media). This mutation is easier to measure than many other ways of assessing mitochondrial flux. The effects of pharmacological modulators on these two measures were consistent, confirming that the high throughput imaging output (autophagosomes co-localizing with mitochondria) reflects mitochondrial quality control [41].

The data generated with these methods suggest that those mitochondria removed by mitophagy are the ones with the highest levels of mutant and the least capacity for oxidative phosphorylation. Given that mitochondrial fusion requires oxidative phosphorylation [51], these mitochondria are likely to be fragmented, and this is another potential signal for mitophagy. mtDNA mutations may also increase ROS production, and increasing ROS damage to mitochondrial proteins and mtDNA could be the signal. PINK1 and Parkin are probably involved in recognizing some types of damage, and ubiquitination is important in many cases. Nevertheless, (1) the type of mitophagy and the trigger required may vary, depending on circumstances (2) when the mutation load or level of damage are very high, mitophagy or other signals may precipitate a cellular catastrophe such as cell-death signalling or mtDNA depletion. Furthermore, the existence of mtDNA disease clearly demonstrates that mitophagy is not able to remove all damaged mitochondria. More work is needed to clarify this.

## MtDNA copy number during pre-implantation development

Whereas relatively few mtDNA genomes (∼200 in mouse) are present in primordial germ cells, the earliest stages of germline differentiation, there is a massive expansion in mtDNA content to 200000–300000 [52] as the oocyte grows and matures. Although oocytes must contain at least 40000–50000 copies of mtDNA in order for an embryo to give rise to a viable foetus [53], unusually high levels of mtDNA at the blastocyst stage of pre-implantation development are associated with failure of implantation [54]. Most authors state that mtDNA content of the oocyte/embryo remains constant between ovulation and morula [55–57], but this is not the case in cows [58] where mtDNA declines by 60% between the 2- and 4/8-cell stage. Immediately after fertilization, mtDNA barely replicates [55–57] and metabolism is slow [59]. However, in pigs there is evidence for mtDNA synthesis between the 2- and 4-cell stage [24]. Hence comparisons with other species must be interpreted with caution. At the blastocyst stage, a proportion of undifferentiated cells become the inner cell mass which eventually gives rise to the body of the embryo, the remainder differentiating into the placenta and membranes. A tiny minority of cells become the precursors of primordial germ cells that transmit mtDNA to future generations.

**Figure 3:**
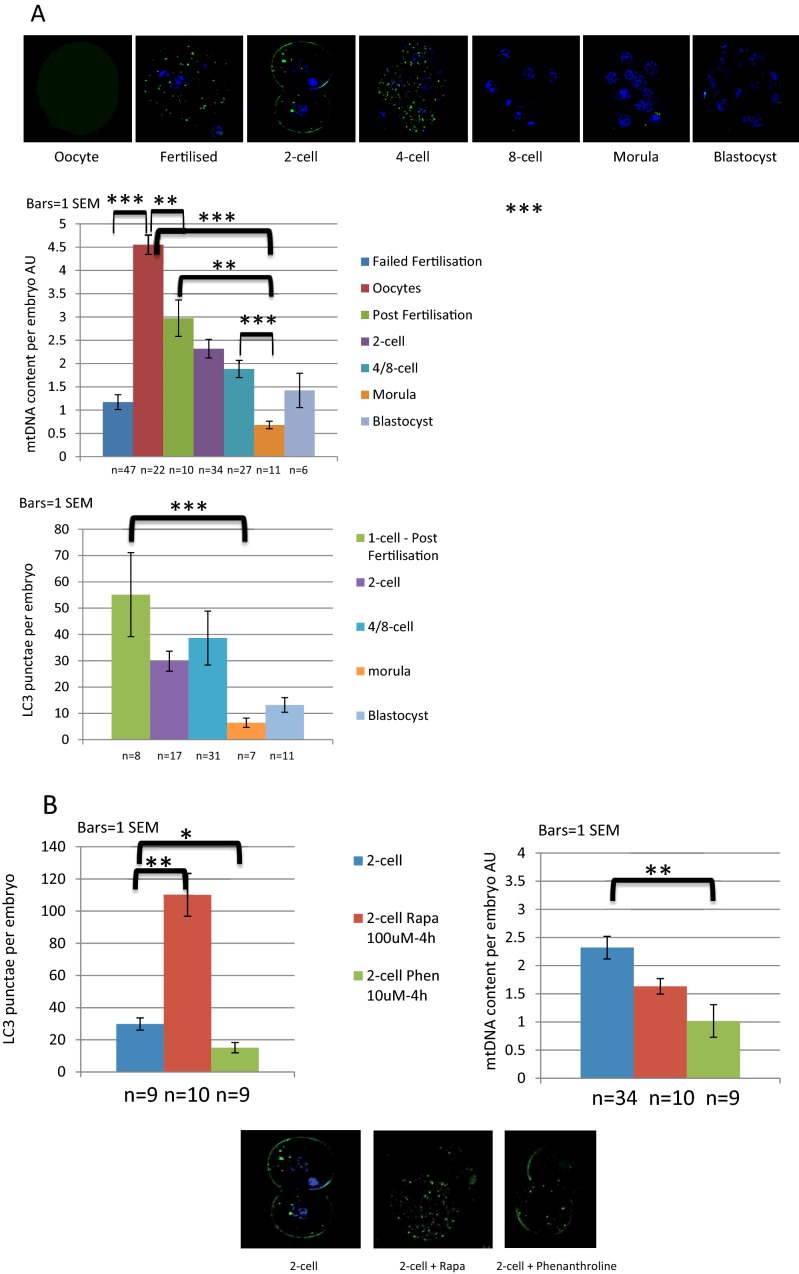
Evidence of autophagy in mouse oocytes and pre-implantation embryos (**A**) Autophagosomes of oocytes and pre-implantation embryos of mice with GFP-tagged LC3 were visualized by fluorescence microscopy and autophagosomes per embryo counted. Relative mtDNA content (AU for arbitrary units) was assessed by single embryo qPCR. The mtDNA of these mice is wild type C57BL/6N and each embryo was individually lysed using an alkaline lysis protocol. After neutralization using tricine, the Taqman quantitative PCR was run for mtDNA along with standards to quantify the mtDNA content per embryo. The results show that mtDNA declines during active autophagy. (**B**) Two-cell embryos have been treated with rapamicin (red bars), phenanthroline (green bars) or not (blue bars) for 4 h to activate mitophagy; rapamycin 100 nM increased autophagosomes number per embryo and mtDNA dropped after exposure to 10 uM phenanthroline. Key *P<0.05, **P<0.01, ***P<0.001 *t* test. *t* tests where distribution was normal, Mann–Whitney elsewhere.

We studied mtDNA copy number in oocytes and pre-implantation embryos generated *in vitro* using quantitative PCR. The mtDNA of these mice is wild type C57BL/6N. Figure 3 shows that the total mtDNA content of the oocyte developing through cleavage stage embryo to blastocyst does not remain constant as described by previous authors [55–57]. Rather, there is a progressive decline in (total per oocyte/embryo) mtDNA to ∼50% by the 4/8-cell stage. The initial drop is apparent in other publications but has barely been reported [60]. Unfertilized oocytes had a higher mtDNA content than either those that failed to fertilize or single cell zygotes. This strongly suggests that mtDNA turns over during IVF development. The reason for the drop in mtDNA content is unclear. However, it could involve mitophagy as discussed below.

Mitochondrial dysfunction drives cells to increase mtDNA copy number in both tissue culture models and *in vivo*, a compensatory adjustment that may be ROS dependent [61]. Both mtDNA content and heteroplasmic load increase with developmental stage in oocytes and cleavage embryos carrying the m.3243G mutant mtDNA [62]: potentially mitochondrial dysfunction drives proliferation of mitochondria and mtDNA as it does in muscle and placenta [63].

## Selection against detrimental mtDNA mutants contributes to the bottleneck

Purifying selection against detrimental mtDNA mutants in mouse [35,36,64] may have evolved to maintain germline homoplasmy. In perhaps the first transmitochondrial mice, we found that mutant mtDNA was rapidly lost [37]. In a second model, maternal transmission of mtDNA rearrangements was attenuated by advancing maternal age [38]. Thirdly, using a proof reading mutation of the mtDNA polymerase to generate multiple mtDNA mutations, Larsson and colleagues demonstrated selection against transmission of deleterious mtDNA mutations to the offspring [35] that was not apparent in oocytes [65,36]. A marked difference in distribution has also been shown between different pathogenic mtDNA in heteroplasmic mutant oocytes, such as m.3243A>G, rearrangements [8] and m.8993T>G [3,12].

Around the onset of biparental gene expression, there is a surge of autophagy [48]. This was neatly demonstrated by Mizushima and colleagues, who visualized autophagosomes by making a mouse in which they tagged the autophagy protein, LC3, with the fluorescent marker, GFP [66,67]. What drives this increased autophagy is unclear. However, this could be a critical stage in purifying selection of transmitted mtDNA. We used Mizushima's mouse to investigate whether the drop in mtDNA we had documented soon after ovulation was accompanied by evidence of autophagy. Figure 3 shows data that autophagosome counts are high at the stage when mtDNA content is dropping. The lowest mtDNA copy number is at the morula stage, by which time the preceding surge in autophagosomes has disappeared.

To determine whether the mtDNA content at this stage could be modulated pharmacologically we exposed two-cell embryos to activators of mitophagy, either rapamycin or phenanthroline for 4 h. We have previously shown that these drugs are able to activate mitophagy in tissue culture cells [41]. However, they appear to be qualitatively and mechanistically different. Rapamycin increases mitophagy by means of its general effect on autophagy, increasing the flux of autophagosomes. In tissue culture cells, we found that rapamycin consistently increases co-localization of mitochondria and autophagosomes, but that there was no effect on overall mtDNA copy number [41]. That rapamycin selects against pathogenic mutations has been demonstrated by others [68], but did not reach statistical significance in our model. Phenanthroline, on the other hand, is a chelator that prevents processing and hence activation of the pro-mitochondrial fusion protein OPA1. Hence phenanthroline causes global mitochondrial fragmentation and this drives mitophagy [69]. It significantly reduces both mitochondrial mass and mtDNA content [41]. Nevertheless the increase in mitophagy appears to be non-selective as it does not improve the load of pathogenic mtDNA in tissue culture.

Figure 3 shows preliminary results of exposing two-cell embryos to each of these drugs, both of which activate mitophagy. As expected, rapamycin significantly increases the number of autophagosomes but has little effect on mtDNA content. Phenanthroline on the other hand decreases the mtDNA content (both *P*<0.01). That phenanthroline does not increase the autophagosome counts is entirely consistent with our tissue culture data where the decrease in mitochondrial mass and mtDNA can be massive [41]. Hence our data suggest that each of these drugs is able to activate mitophagy in two-cell embryos. This is intriguing, but more work is needed both to confirm it, and to determine whether either of these drugs would be selective against mutant mtDNA.

A theoretical framework with which to analyse and predict the effects of these experimental interventions has recently been developed. Johnston et al. [6] combined mathematical modelling with a range of existing and new experimental measurements of heteroplasmy during development to quantitatively describe the processes altering developing mtDNA populations in mice. The theory, supported by existing and new experiments, predicts that interventions increasing mitophagy will both exacerbate any selection against detrimental mutants and increase the power of the developmental bottleneck to increase heteroplasmy variance between oocytes. These arise because increased mitophagy must either decrease the size of cellular mtDNA populations or provoke a compensatory increase in mtDNA replication to stabilize copy number. Both of these outcomes strengthen the effects of selection and random drift, either due to a smaller population size or due to an increase in the rates of the underlying cellular processes. The relative importance of random drift and selection can be accounted for by this theory. This mathematical treatment reinforces preliminary experimental findings suggesting that mitophagy activators like rapamycin or phenanthroline may constitute new axes of intervention to increase the power of mtDNA bottlenecking and selection to decrease mutant load.

## Conclusions

We have developed methods for investigating mitophagy based on high throughput imaging in order to understand mtDNA segregation in heteroplasmic mitochondrial disease. We showed that mitophagy can be increased in cultures from patients with the mtDNA mutation, m.3243A>G, by energetic stress and by drug modulators of mitophagy. We studied mitophagy during pre-implantation development in the mouse, obtaining data that is consistent with autophagic activity that results in a drop in mtDNA copy number soon after ovulation. Preliminary data suggest that mtDNA copy number may be driven both by mitochondrial dysfunction and by drug modulators of mitophagy. Theoretical treatments based on mathematical modelling and data-drive statistical analysis support the idea that increasing mitophagy may help to robustly remove mutant mtDNA. More data are needed to inform regulators of mitochondrial replacement therapy about these important processes that may determine its success or failure.

## Abbreviations

IVF: *in vitro* fertilization
MRT: mitochondrial replacement therapy
PGD: pre-implantation genetic diagnosis
ROS: reactive oxygen species
SIMH: stress-induced mitochondrial hyper-fusion
TALEN: transcription activator-like effector nuclease
